# Supplementary material for: Advancing workpiece dimension measurement: Integrating AI-based edge detection with machine vision and coordinate measuring systems
Source: PLoS One. 2026 Mar 23;21(3):e0342797. doi: 10.1371/journal.pone.0342797 (PMC13008250; doi:10.1371/journal.pone.0342797)
Supplement: S1 File — (DOCX) [file pone.0342797.s001.docx]

**S 1. File
CODE OF CREATING IMAGE ANALYSIS AND DETECTION**

import cv2

import numpy as np

def process_image(image_path):

*# Read input image*

original = cv2.imread(image_path)

if original is None:

print("Error: Image not found")

return

*# Create processing windows*

cv2.namedWindow('Parameters')

cv2.namedWindow('Processing Pipeline')

cv2.resizeWindow('Parameters', 600, 400)

cv2.resizeWindow('Processing Pipeline', 1000, 600)

*# Initialize parameters with default values*

params = {

'gauss_kernel': 5,

'canny_th1': 100,

'canny_th2': 200,

'hough_dp': 1,

'hough_minDist': 30,

'hough_param1': 100,

'hough_param2': 30,

'hough_minRadius': 10,

'hough_maxRadius': 100

}

*# Create trackbars for parameter adjustment*

cv2.createTrackbar('Gaussian Kernel', 'Parameters', params['gauss_kernel'], 30, lambda x: None)

cv2.createTrackbar('Canny Thresh1', 'Parameters', params['canny_th1'], 500, lambda x: None)

cv2.createTrackbar('Canny Thresh2', 'Parameters', params['canny_th2'], 500, lambda x: None)

cv2.createTrackbar('Hough dp', 'Parameters', params['hough_dp'], 20, lambda x: None)

cv2.createTrackbar('Hough minDist', 'Parameters', params['hough_minDist'], 200, lambda x: None)

cv2.createTrackbar('Hough param1', 'Parameters', params['hough_param1'], 200, lambda x: None)

cv2.createTrackbar('Hough param2', 'Parameters', params['hough_param2'], 100, lambda x: None)

cv2.createTrackbar('Min Radius', 'Parameters', params['hough_minRadius'], 200, lambda x: None)

cv2.createTrackbar('Max Radius', 'Parameters', params['hough_maxRadius'], 500, lambda x: None)

while True:

*# Get current trackbar values*

params['gauss_kernel'] = max(1, cv2.getTrackbarPos('Gaussian Kernel', 'Parameters') | 1) *# Ensure odd number*

params['canny_th1'] = cv2.getTrackbarPos('Canny Thresh1', 'Parameters')

params['canny_th2'] = cv2.getTrackbarPos('Canny Thresh2', 'Parameters')

params['hough_dp'] = max(1, cv2.getTrackbarPos('Hough dp', 'Parameters'))

params['hough_minDist'] = cv2.getTrackbarPos('Hough minDist', 'Parameters')

params['hough_param1'] = cv2.getTrackbarPos('Hough param1', 'Parameters')

params['hough_param2'] = cv2.getTrackbarPos('Hough param2', 'Parameters')

params['hough_minRadius'] = cv2.getTrackbarPos('Min Radius', 'Parameters')

params['hough_maxRadius'] = cv2.getTrackbarPos('Max Radius', 'Parameters')

*# Convert to grayscale*

gray = cv2.cvtColor(original, cv2.COLOR_BGR2GRAY)

*# Apply Gaussian blur*

blurred = cv2.GaussianBlur(gray, (params['gauss_kernel'], params['gauss_kernel']), 0)

*# Canny edge detection*

edges = cv2.Canny(blurred, params['canny_th1'], params['canny_th2'])

*# Hough circle detection*

circles = cv2.HoughCircles(

edges,

cv2.HOUGH_GRADIENT,

dp=params['hough_dp'],

minDist=params['hough_minDist'],

param1=params['hough_param1'],

param2=params['hough_param2'],

minRadius=params['hough_minRadius'],

maxRadius=params['hough_maxRadius']

)

*# Create visualization pipeline*

vis_gray = cv2.cvtColor(gray, cv2.COLOR_GRAY2BGR)

vis_blur = cv2.cvtColor(blurred, cv2.COLOR_GRAY2BGR)

vis_edges = cv2.cvtColor(edges, cv2.COLOR_GRAY2BGR)

result = original.copy()

*# Draw detected circles*

if circles is not None:

circles = np.uint16(np.around(circles))

for i in circles[0, :]:

*# Draw outer circle*

cv2.circle(result, (i[0], i[1]), i[2], (0, 255, 0), 2)

*# Draw center*

cv2.circle(result, (i[0], i[1]), 2, (0, 0, 255), 3)

*# Create processing pipeline visualization*

pipeline_top = np.hstack([original, vis_gray, vis_blur])

pipeline_bottom = np.hstack([vis_edges, result, result]) *# Extra space for labels*

pipeline = np.vstack([pipeline_top, pipeline_bottom])

*# Add labels*

labels = [

"Original Image",

"Grayscale Conversion",

f"Gaussian Blur (k={params['gauss_kernel']})",

"Canny Edge Detection",

"Circle Detection Result",

"Parameter Adjustment"

]

positions = [(10, 30), (330, 30), (650, 30),

(10, 360), (330, 360), (650, 360)]

for (x, y), label in zip(positions, labels):

cv2.putText(pipeline, label, (x, y),

cv2.FONT_HERSHEY_SIMPLEX, 0.7, (0, 0, 255), 2)

*# Display results*

cv2.imshow('Processing Pipeline', pipeline)

*# Check for exit key*

key = cv2.waitKey(1) & 0xFF

if key == 27 or key == ord('q'): *# ESC or 'q' to quit*

break

cv2.destroyAllWindows()

--------------------------------------------------------------------------------------------------------------------------------------------------------

**# Creating the dense Code using CV with library as TensorFlow**

import numpy as np

import matplotlib.pyplot as plt

import seaborn as sns

import tensorflow as tf

from tensorflow.keras.applications import EfficientNetB0

from tensorflow.keras.models import Model

from tensorflow.keras.layers import Dense, GlobalAveragePooling2D, Dropout

from tensorflow.keras.optimizers import Adam

from tensorflow.keras.preprocessing.image import ImageDataGenerator

from sklearn.metrics import confusion_matrix, classification_report, roc_curve, auc

from time import time

*# Configuration*

IMG_SIZE = (224, 224)

BATCH_SIZE = 16

EPOCHS = 20

NUM_CLASSES = 2

CLASS_NAMES = ['Model 1', 'Model 2']

*# Prepare data generators*

train_datagen = ImageDataGenerator(

rescale=1./255,

validation_split=0.2,

rotation_range=15,

zoom_range=0.1,

horizontal_flip=True

)

test_datagen = ImageDataGenerator(rescale=1./255)

*# Replace with your dataset path*

train_generator = train_datagen.flow_from_directory(

'path/to/delrin_dataset/train',

target_size=IMG_SIZE,

batch_size=BATCH_SIZE,

class_mode='categorical',

subset='training',

shuffle=True

)

val_generator = train_datagen.flow_from_directory(

'path/to/delrin_dataset/train',

target_size=IMG_SIZE,

batch_size=BATCH_SIZE,

class_mode='categorical',

subset='validation',

shuffle=False

)

test_generator = test_datagen.flow_from_directory(

'path/to/delrin_dataset/test',

target_size=IMG_SIZE,

batch_size=BATCH_SIZE,

class_mode='categorical',

shuffle=False

)

*# Create EfficientNet model with transfer learning*

base_model = EfficientNetB0(

include_top=False,

weights='imagenet',

input_shape=(IMG_SIZE[0], IMG_SIZE[1], 3)

)

*# Freeze base layers*

base_model.trainable = False

*# Build classification head*

x = base_model.output

x = GlobalAveragePooling2D()(x)

x = Dense(256, activation='relu')(x)

x = Dropout(0.3)(x)

predictions = Dense(NUM_CLASSES, activation='softmax')(x)

model = Model(inputs=base_model.input, outputs=predictions)

*# Compile model*

model.compile(

optimizer=Adam(learning_rate=0.0001),

loss='categorical_crossentropy',

metrics=['accuracy']

)

*# Train model*

history = model.fit(

train_generator,

steps_per_epoch=train_generator.samples // BATCH_SIZE,

validation_data=val_generator,

validation_steps=val_generator.samples // BATCH_SIZE,

epochs=EPOCHS

)

*# Evaluate model*

test_loss, test_acc = model.evaluate(test_generator)

print(f'\nTest accuracy: {test_acc:.4f}, Test loss: {test_loss:.4f}')

*# Generate predictions*

y_pred = model.predict(test_generator)

y_pred_classes = np.argmax(y_pred, axis=1)

y_true = test_generator.classes

*# Generate classification report*

print("\nClassification Report:")

print(classification_report(y_true, y_pred_classes, target_names=CLASS_NAMES))

*# Confusion matrix*

cm = confusion_matrix(y_true, y_pred_classes)

*# Performance Visualization*

plt.figure(figsize=(15, 12))

*# 1. Confusion Matrix*

plt.subplot(2, 2, 1)

sns.heatmap(cm, annot=True, fmt='d', cmap='Blues',

xticklabels=CLASS_NAMES, yticklabels=CLASS_NAMES)

plt.title('Confusion Matrix', fontsize=14)

plt.xlabel('Predicted', fontsize=12)

plt.ylabel('Actual', fontsize=12)

*# 2. ROC Curve*

plt.subplot(2, 2, 2)

fpr, tpr, _ = roc_curve(y_true, y_pred[:, 1])

roc_auc = auc(fpr, tpr)

plt.plot(fpr, tpr, color='darkorange', lw=2,

label=f'ROC curve (area = {roc_auc:.2f})')

plt.plot([0, 1], [0, 1], color='navy', lw=2, linestyle='--')

plt.xlim([0.0, 1.0])

plt.ylim([0.0, 1.05])

plt.xlabel('False Positive Rate', fontsize=12)

plt.ylabel('True Positive Rate', fontsize=12)

plt.title('Receiver Operating Characteristic', fontsize=14)

plt.legend(loc="lower right")

*# 3. Accuracy History*

plt.subplot(2, 2, 3)

plt.plot(history.history['accuracy'], label='Training Accuracy')

plt.plot(history.history['val_accuracy'], label='Validation Accuracy')

plt.title('Training and Validation Accuracy', fontsize=14)

plt.xlabel('Epochs', fontsize=12)

plt.ylabel('Accuracy', fontsize=12)

plt.legend()

*# 4. Loss History*

plt.subplot(2, 2, 4)

plt.plot(history.history['loss'], label='Training Loss')

plt.plot(history.history['val_loss'], label='Validation Loss')

plt.title('Training and Validation Loss', fontsize=14)

plt.xlabel('Epochs', fontsize=12)

plt.ylabel('Loss', fontsize=12)

plt.legend()

plt.tight_layout()

plt.savefig('delrin_classification_results.png', dpi=300)

plt.show()
