# Supplementary material for: Advancing workpiece dimension measurement: Integrating AI-based edge detection with machine vision and coordinate measuring systems
Source: PLoS One. 2026 Mar 23;21(3):e0342797. doi: 10.1371/journal.pone.0342797 (PMC13008250; doi:10.1371/journal.pone.0342797)
Supplement: S2 File — (DOCX) [file pone.0342797.s002.docx]

**S 2. FILE**

**STATISTICAL ANALYSIS**

**Table of Reliability**

| Cronbach's Alpha | Cronbach's Alpha Based on Standardized Items | N of Items |
| --- | --- | --- |
| **.999** | .999 | 3 |

**Table of Correlations**

|  | | Measure VI | Measure CMM | Measure CNN |
| --- | --- | --- | --- | --- |
| Measure VI | Pearson Correlation | 1 | **.996^**^** | **.995^**^** |
|  | Sig. (2-tailed) |  | **.000** | **.000** |
|  | N | 60 | 60 | 60 |
| Measure CMM | Pearson Correlation | **.996^**^** | 1 | **1.000^**^** |
|  | Sig. (2-tailed) | **.000** |  | **.000** |
|  | N | 60 | 60 | 60 |
| Measure CNN | Pearson Correlation | **.995^**^** | **1.000^**^** | 1 |
|  | Sig. (2-tailed) | **.000** | **.000** |  |
|  | N | 60 | 60 | 60 |

| ****. Correlation is significant at the 0.01 level (2-tailed).** |
| --- |

**One-Way ANOVA**

| **Table of Test of Homogeneity of Variances** | | | | | | | | | |  |  |
| --- | --- | --- | --- | --- | --- | --- | --- | --- | --- | --- | --- |
|  | | Levene Statistic | | df1 | | df2 | | Sig. | |  |  |
| Measure VI | | 1.724 | | 4 | | 55 | | .158 | |  |  |
| Measure CMM | | 2.039 | | 4 | | 55 | | .102 | |  |  |
| Measure CNN | | .166 | | 4 | | 55 | | .955 | |  |  |
| **Table of One-Way ANOVA** | | | | | | | | | | |  |
|  | | | Sum of Squares | | df | | Mean Square | | F | | Sig. |
| Measure VI | Between Groups | | 774.122 | | 4 | | 193.530 | | 15635625.390 | | **.000** |
|  | Within Groups | | .001 | | 55 | | .000 | |  | |  |
|  | Total | | 774.122 | | 59 | |  | |  | |  |
| Measure CMM | Between Groups | | 757.648 | | 4 | | 189.412 | | 72146966.569 | | **.000** |
|  | Within Groups | | .000 | | 55 | | .000 | |  | |  |
|  | Total | | 757.648 | | 59 | |  | |  | |  |
| Measure CNN | Between Groups | | 761.289 | | 4 | | 190.322 | | 345146583.475 | | **.000** |
|  | Within Groups | | .000 | | 55 | | .000 | |  | |  |
|  | Total | | 761.289 | | 59 | |  | |  | |  |

**Means Plots**


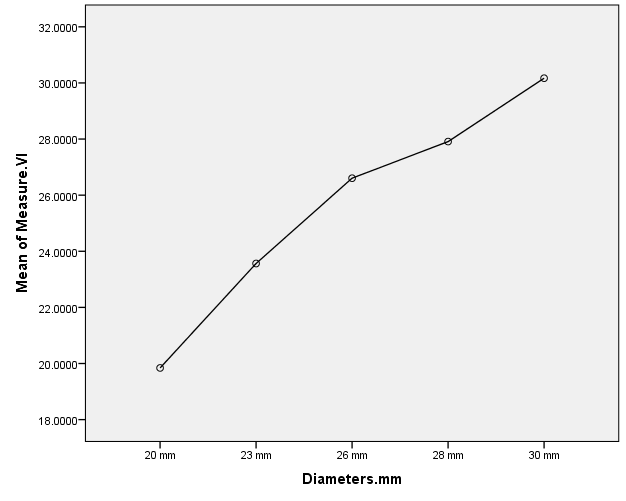


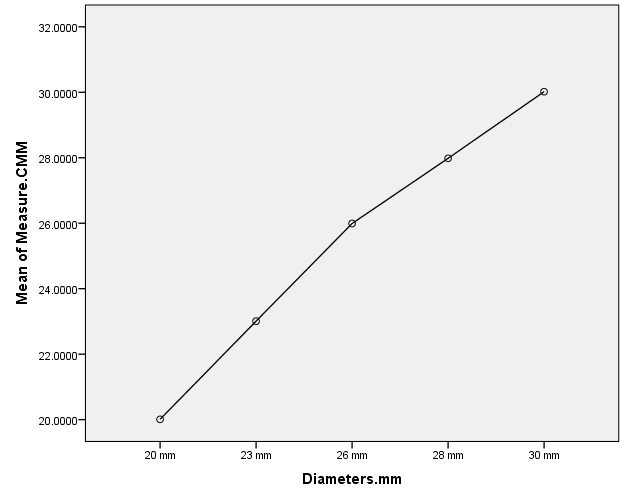


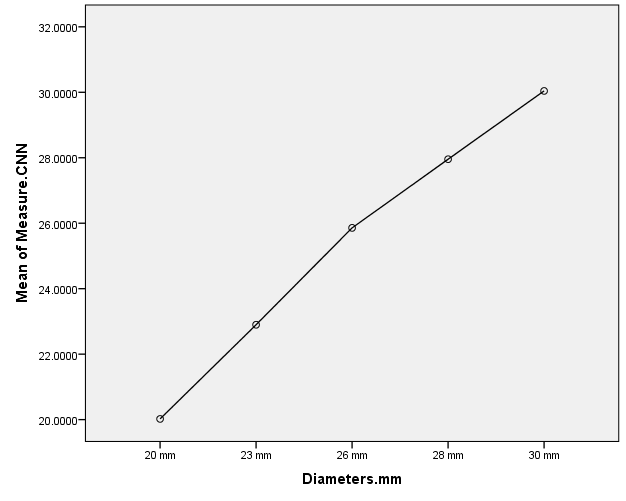


**Descriptive**

**(Table 1) of Descriptive**

|  | N | Min. | Max. | Mean | Std. Error | Std. Deviation | Variance | | |
| --- | --- | --- | --- | --- | --- | --- | --- | --- | --- |
| Measure.VI | 60 | 19.836 | 30.170 | 25.618  25.402  25.355 | .4676 | 3.622 | 13.121 | | |
| Measure CMM | 60 | 20.006 | 30.021 |  | .4626 | 3.584 | 12.841 | | |
| Measure CNN | 60 | 20.020 | 30.042 |  | .4637 | 3.592 | 12.903 | | |
|  |  |  |  |  |  |  | |  |  |
